# On-Chip DNA Assembly via Dielectrophoresis

**DOI:** 10.3390/mi16010076

**Published:** 2025-01-11

**Authors:** Xichuan Rui, Lin-Sheng Wu, Xin Zhao

**Affiliations:** State Key Laboratory of Radio Frequency Heterogeneous Integration, Shanghai Jiao Tong University, Shanghai 200240, China; ruixichuan@sjtu.edu.cn (X.R.); wallish@sjtu.edu.cn (L.-S.W.)

**Keywords:** gene synthesis, gene assembly, dielectrophoresis, on-chip, helical forked electrode

## Abstract

On-chip gene synthesis has the potential to improve the synthesis throughput and reduce the cost exponentially. While there exist several microarray-based oligo synthesis technologies, on-chip gene assembly has yet to be demonstrated. This work introduces a novel on-chip DNA assembly method via dielectrophoresis (DEP) that can potentially be integrated with microarray-based oligo synthesis on the same chip. Our DEP chip can selectively manipulate oligos and guide their movement without perturbing the surrounding fluid medium, thus aiding in DNA assembly. Helical forked electrode design has been optimized for compatibility with DEP, ensuring efficient control over target oligos. By applying an alternating current signal set at 2 MHz, we successfully achieve the desired directed movement of oligonucleotides. Additionally, chemical treatments combined with photoirradiation enabled the connection of complementary gene sequences and the subsequent release of single-stranded DNA products. Sequencing results validate the effective assembly of DNA fragments, approximately 500 base pairs in length, using our DEP device.

## 1. Introduction

Gene synthesis is one of the foundational technologies for modern molecular biology, crucial for synthetic biology, therapeutics, diagnostics, DNA information storage, and many other fields [1,2]. Compared to gene sequencing, the cost has decreased exponentially over the past two decades; gene synthesis has progressed at a much slower pace [3]. The discrepancy between the throughput/cost of DNA synthesis and sequencing remains a major bottleneck in molecular biology research [1,3].

The synthesis of a gene usually begins with the design and chemical synthesis of overlapping oligonucleotide (oligo) sequences. Gene fragments are then assembled from these oligos to obtain longer sequences via several methods, including Gibson assembly and Golden Gate cloning [1,4]. Recently, several high-throughput oligo synthesis technologies have been developed leveraging semiconductor technology, enabling exponential decay in cost per oligo. Thousands and even millions of oligos can be synthesized simultaneously and independently on silicon chips with miniscule chemical reagents [5,6]. Given roughly constant reagents needed per chip area, the cost per oligo is inversely proportional to the synthesis throughput, analogous to Moore’s law. However, gene synthesis costs remain high because gene assembly, purification, and subsequent steps are not miniaturized on chips. High-throughput gene assembly on-chip and further integration with oligo synthesis on the same chip can potentially reduce gene synthesis costs by orders of magnitude.

On-chip electrowetting has been investigated for DNA manipulation by controlling the wetting properties of microchannel surfaces [7,8,9,10]. By adjusting the electric field intensity and polarity on the microchannel surface, directed movement and assembly of DNA fragments can be achieved. However, electrowetting requires special surface treatment for precise control of welling behaviour [11], which is incompatible with on-chip oligo synthesis. In contrast, our DEP-based platform offers a simpler and more flexible solution that eliminates the need for surface modifications, making it highly compatible with existing on-chip systems. DEP enables precise manipulation of nanoparticles under nonuniform electric fields and allows for the integration of oligo synthesis and gene assembly on the same chip without requiring special surface treatments [12,13,14].

Gene assembly via DEP has yet to be demonstrated. In this study, we introduce for the first time a novel one-pot gene assembly method using a DEP chip, paving the way for on-chip integration of oligo synthesis and gene assembly. The precise control of the electric field within the helical forked electrode array enables selective and reliable manipulation of oligos. Traditional well-plate methods for DNA assembly require larger reaction volumes and longer times, with significant reagent consumption. To address these limitations, we introduce a DNA assembly chip platform based on DEP, which uses electric fields to precisely manipulate DNA molecules for efficient assembly with minimal reagent use. Unlike traditional well-plate methods, the DEP platform achieves DNA assembly in significantly smaller reaction volumes, reducing reagent consumption and shortening reaction times. By generating uniform DEP forces across multiple electrodes, the DEP chip has the potential to scale up for high-throughput applications. This scalability, combined with the platform’s precision in manipulating DNA molecules, provides a transformative approach for miniaturized and high-throughput gene synthesis.

## 2. Results

### 2.1. DNA Enrichment and Transportation Using the DEP System

**Figure 1:**
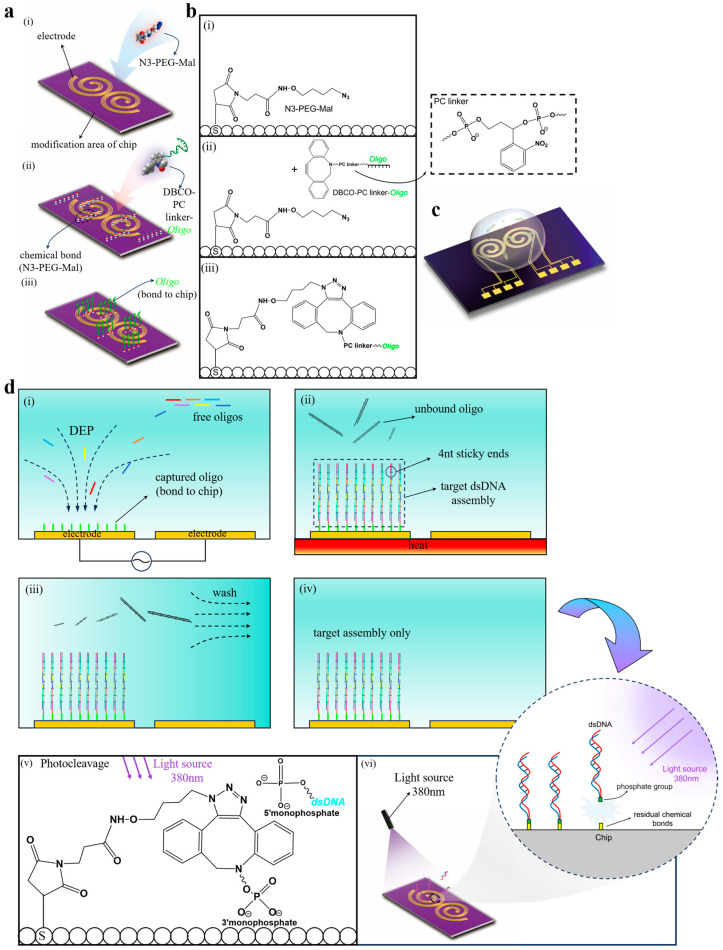
Schematic of the DEP device for gene synthesis. (**a**) The chemical modification of the DEP electrode allows DBCO-PC linker-Oligos to bond to the chip. (**b**) N3-PEG-Mal reacts with Oligos on the electrode surface, resulting in the formation of Oligos. (**c**) Oligos manipulation occurs on the electrode surface through DEP forces. (**d**) By applying an AC signal, complementary pairing takes place between the free oligos and the bonded oligos on the electrode. Unpaired oligos are washed away, and controlled light exposure facilitates the release of the target dsDNA, ultimately producing the desired target dsDNA.

Here, we consider a helical forked electrode, as shown in Figure 1. The electrodes are designed as four parallelly arranged sub-electrodes, with each sub-electrode individually controlled by a distinct electrical signal. By applying alternating current (AC) signals, DEP forces are generated on the chip, allowing for the manipulation of nano-sized oligo. This process propels the oligo towards adjacent electrodes. For the long-distance transportation of oligo, an alternating application of electrical signals is employed. This creates DEP forces facilitating the trans-electrode transfer of oligo across electrode arrays.

Surface chemical modification of the electrodes is critical for oligo movement on the chip, as depicted in Figure 1a. The electrodes undergo cleaning with acetone to remove debris and photoresist remnants from the silicon wafer surface. The chip is then subjected to air plasma treatment for five minutes at 75 Pa and 180 W, revealing silicon-oxygen bonds (Si-OH) on the surface. A mixture of methylphenylsiloxane resin (MPS), acetic acid, and toluene is prepared and shaken with the chip for ten hours. The chip is subsequently reacted with N3-PEG-Mal, culminating in the final modification with DBCO-PC Linker CE-ssDNA.

Following chemical modification, AC signals are applied as shown in Figure 1b–d, allowing pre-split complementary sequence oligos to form complementary keys on the DEP electrode (refer to Table S1 for the sequence). Unpaired oligos are washed away, and controlled light exposure releases the target dsDNA, yielding the desired dsDNA.

We assessed the oligos enrichment capabilities of our electrode array using an oligos solution and an alternating current power supply (2 MHz, 50 Vrms). The electrode width and distance between sub-electrodes were set at 25 μm, with an oligos concentration of 20 ng/μL.

**Figure 2:**
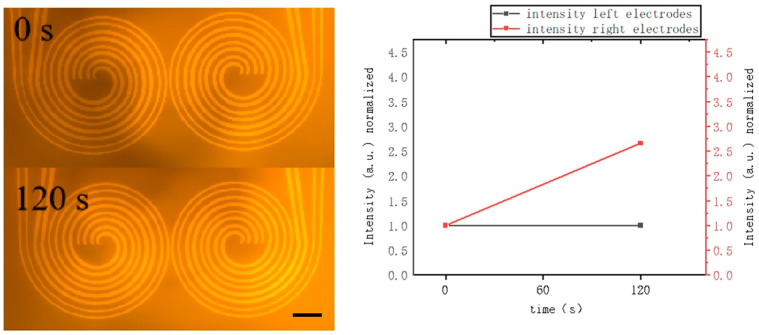
Experimental observation of DNA enrichment on the right electrode. The experiment was conducted under an alternating current power supply (2 MHz, 50 Vrms). The electrode width is 25 µm, and the distance between sub-electrodes is 25 μm, corresponding to a DNA concentration of 20 ng/µL. The data results have been treated with normalization (Scale bar: 200 μm).

Experimental observations, as captured in Figure 2 and Supplementary Information S1 (video), indicate that when the helical forked electrode on the right is actuated, DEP forces propel oligos toward the active electrode, resulting in oligos enrichment. Utilizing this principle, we also demonstrated the movement of oligos between two electrodes. The electrode width was 25 μm, the distance between sub-electrodes was 25 μm, and the gap between two helical forked electrodes was 120 μm. The oligos begin to accumulate on the right electrode when actuated, as shown in Figure 2 (from Movie S1 in Supplementary Information). The fluorescence intensity on this electrode increases from 1 to 4.2 under the influence of AC-DEP while remaining nearly constant without AC-DEP. Conversely, the fluorescence intensity on the left electrode initially remains at approximately 1 and then gradually increases to 3.7 within 200 s as DEP forces shift oligos from the right to the left electrode. This transport effect is confirmed by analyzing fluorescence data from the video, demonstrating that oligos can be effectively transferred across electrodes in our device.

**Figure 3:**
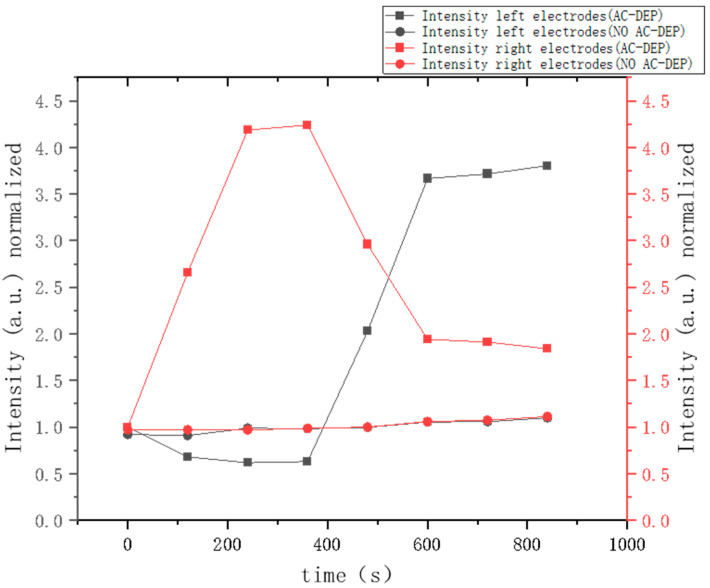
The graph depicts the change in fluorescence intensity over time when DNA is driven by DEP forces. An AC signal was applied to the right electrode, leading to a significant enhancement in the fluorescence signal on the right side. Upon switching the AC signal to the left electrode, a noticeable increase in the signal on the left side occurred, accompanied by a concurrent decrease in the fluorescence signal on the right side.

Figure 3 presents a line chart depicting fluorescence intensity changes over time. The red square-marked curve indicates the fluorescence intensity on the right electrode group with AC-DEP applied, while the red circle-marked curve shows the intensity without AC-DEP. The black square-marked curve represents the fluorescence intensity on the left electrode group with AC-DEP. Notably, the application of an AC signal on the right electrode side markedly raises the oligos fluorescence signal intensity compared to the unexcited state. Switching the signal from the right to the left electrode results in a discernible reduction in fluorescence on the right and a corresponding increase on the left, confirming the directed movement of oligos between electrodes.

### 2.2. Simulation Model Description

**Figure 4:**
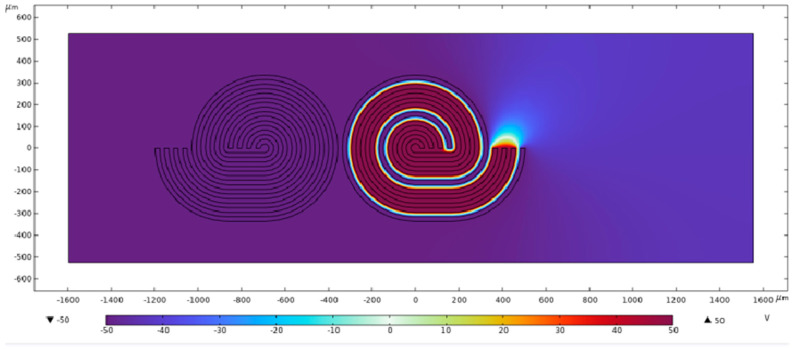
COMSOL Multiphysics^®^ software is employed for simulating the DEP electrodes, with specific parameters allowing individual control of the electrode at a frequency of 2 MHz. This enables the polarization and manipulation of DNA on the electrode.

To evaluate oligo manipulation within an actual DEP system, we first conducted a simulation analysis by using COMSOL Multiphysics^®^ software V 5.4. Figure 4 demonstrates that when voltage is applied to the first electrode through an alternating electric field (see Figure 4, our helical forked electrodes can independently control a single electrode of 25 microns in width. When voltage is applied to both the first and third electrodes (Figure 4, it enables independent control of two separate electrodes, each 25 microns wide, polarizing the oligos and generating DEP forces.

### 2.3. Gene Assembly by DEP Manipulation

**Figure 5:**
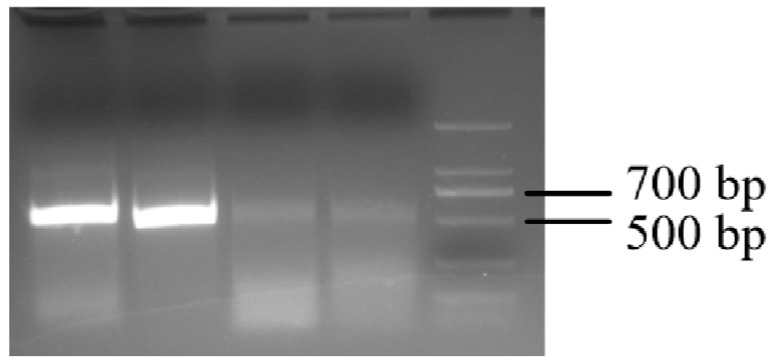
Gel electrophoresis image of 2% agarose gel for double-stranded DNA. The DNA was obtained through electrophoresis-driven complementary pairing of single-stranded DNA, indicating that the length of the DNA is approximately in the 500 bp range.

Employing the DEP electrode-based platform described earlier, we can manipulate and concentrate oligos at specific sites for gene synthesis. The split gene sequence used is listed in Table S1. A chip with a helical forked electrode surface modified with DBCO-PC Linker CE-ssDNA was prepared through chemical treatment. By applying AC-DEP, we directed complementary oligos in solution to the electrode surface modified with DBCO-PC Linker CE-ssDNA. UV light exposure at 15 cm (365 nm emission peak, 300 nm cut-off, ~31 cm intensity at 1.1 mW) severed the PC-linker, freeing the complementary genes for recovery. PCR amplification yielded complete double-stranded products. Following PCR, 50 mL of the product underwent agarose gel electrophoresis and gel extraction alongside 5 mL of 2K DNA Marker. The extracted samples were ligated, and single colonies were sequenced. The sequencing results, as shown in Figure 5 and detailed in Supplementary Information Table S1, indicated the successful synthesis of approximately 500 bp of DNA and provided good sequencing data from the ssDNA.

## 3. Discussion

We utilize DEP technology to manipulate oligos and conduct on-chip DNA assembly. We introduce a novel single-step gene assembly approach with helical forked electrode arrays, allowing for precise control of the internal electric fields. This DEP chip enables selective manipulation of oligos, directing their movement without disturbing the surrounding liquid, thereby facilitating DNA assembly. The electrode design is optimized for compatibility with DEP, ensuring effective control of target oligos regardless of the sizes. By applying an alternating current signal at 2 MHz, we achieved the desired oligonucleotide movement. Furthermore, chemical treatments combined with photoirradiation enabled the connection of complementary gene sequences and the subsequent release of single-stranded DNA products. Sequencing results validate the effective assembly of DNA fragments, approximately 500 base pairs in length, using our DEP device. This study not only achieves on-chip DNA assembly based on DEP technology but also holds promise for integrating oligos synthesis and DNA assembly on the same chip.

In this study, our proposed DEP-based DNA assembly platform demonstrated significant advantages, particularly in reagent consumption and reaction time. Compared to traditional well-plate methods, our DEP platform achieved efficient DNA assembly in smaller reaction volumes and significantly reduced reagent consumption. Experimental results show that we successfully assembled a 500 bp DNA fragment using microliter volumes of reagents, which not only reduced material costs but also minimized chemical waste.

In terms of reaction time, the DEP technology outperforms traditional methods. Conventional well-plate methods typically require 2–3 h to assemble a 500 bp DNA construct, whereas our DEP platform reduces this time to under 30 min, saving over 75% of the time. This improvement highlights that DEP not only increases efficiency but also provides greater potential for high-throughput DNA assembly.

Furthermore, compared to electrowetting technology, DEP offers greater operational flexibility. Electrowetting typically relies on precise surface chemical modifications and requires specific hydrophobic droplets to control reagent flow, adding complexity to the process. In contrast, our DEP platform requires no complex surface modifications, making it easier to operate while achieving faster assembly reactions, thus improving experimental efficiency. As a result, DEP demonstrates clear advantages in reagent consumption, reaction time, and ease of operation compared to traditional methods and electrowetting technology, showcasing its potential for gene synthesis miniaturization and high-throughput applications.

The simulation results show that applying an alternating current signal at 2 MHz generates sufficient DEP forces around each electrode. This allows for the precise concentration and polarization of the oligos, guiding them toward the electrodes where they can be captured and assembled. The simulation analysis of the electric fields further confirms the effectiveness of the helical forked electrode design, which ensures that the DEP forces are sufficiently strong to manipulate oligos of various sizes, regardless of their specific dimensions.

Measures for managing Joule heating were implemented in the DEP chip design, and no significant heat dissipation was observed during the experiments. The temperature increase remained minimal and did not interfere with the DNA assembly process. Additionally, to address the potential issue of solvent evaporation, we incorporated evaporation prevention strategies. The design of the microfluidic chambers helped to effectively minimize solvent loss, ensuring that the volume of the solution remained stable throughout the reaction. These measures collectively maintained the stability of the experimental conditions, which is crucial for the precision and reliability of the DNA assembly process.

We also acknowledge the limitations of our current system. Although the DEP platform performs efficiently at small scales, scaling up for high-throughput or large-scale applications remains a challenge. Additionally, the platform’s sensitivity to external factors, such as temperature and ionic strength, may affect the precision of oligo manipulation. Future work will focus on refining the chip design and improving the robustness of the system for broader applications.

## 4. Materials and Methods

### 4.1. Dielectrophoresis

DEP has been established as highly effective for the manipulation of biological samples [15,16,17]. It operates by exerting a force through controlled frequency and voltage across electrodes [18,19]. An entity in an asymmetric electric field forms dipoles that experience an asymmetric effect. The resultant force, known as the DEP force, is described by the equation:

(1) $$F_{DEP}=2\pi r^{3}\varepsilon_{m}Re\left\{F_{CM}\right\}\nabla E^{2}$$

r is the effective radius of the entity, $\varepsilon_{m}$ is the dielectric constant of the medium, E is the root mean square value of the electric field, $F_{CM}$ is the real part of the Clausius-Mossotti (CM) factor, which determines the sign of the DEP force and the direction of particle motion, is described by the equation:

(2) $$F_{CM}=\frac{\varepsilon_{p}^{*}-\varepsilon_{m}^{*}}{\varepsilon_{p}^{*}+2\varepsilon_{m}^{*}}$$

$\varepsilon_{p}^{*}$ and $\varepsilon_{m}^{*}$ describe the complex dielectric constants of the entity and the fluid medium in which it is suspended, respectively, and are given by:

(3) $$\varepsilon^{*}=\varepsilon -j\frac{\sigma }{\omega }$$

Among them, ε is the dielectric constant, σ is the conductivity, j is the imaginary unit, and ω is the angular frequency. $F_{CM}$ determines the sign of the DEP force and the direction of particle motion. Particles with different dielectric properties experience different DEP forces depending on the frequency applied to the electrodes. The real part of $F_{CM}$ is in the range of −0.5 to 1. Positive $Re\left\{F_{CM}\right\}$ will cause particles to develop a positive DEP force (pDEP) and push them towards high electric field regions. In this case, the particles exhibit more polarizability than the fluid medium.

### 4.2. Sample Preparation

The oligos used in this study (listed in Table S1) were synthesized by SiBio Co., Ltd. (Suzhou, China) with high-performance liquid chromatography (HPLC) purification. Other materials, including T4 DNA ligase, Exonuclease III, Phi29 DNA polymerase, and various buffers, were sourced from New England Biolabs (Beverly, MA, USA). A deoxynucleotide triphosphate (dNTP) solution mixture was purchased from Generay Biotech Co., Ltd. (Shanghai, China). Oligos modified with a dibenzocyclooctyne (DBCO) group and a PC Linker CE (DBCO-PC Linker CE-ssDNA) were synthesized by SiBio Co., Ltd. (Suzhou, China). Agarose was purchased from Baygene Biotech Co., Ltd. (Beijing, China). A DNA marker was purchased from Sangon Biotech Synthesis Co., Ltd. (Shanghai, China). Other reagents were all of analytical grade or higher quality. All solutions were prepared with Milli-Q water (Millipore, Waltham, MA, USA).

### 4.3. Fabrication of the DEP Device

**Figure 6:**
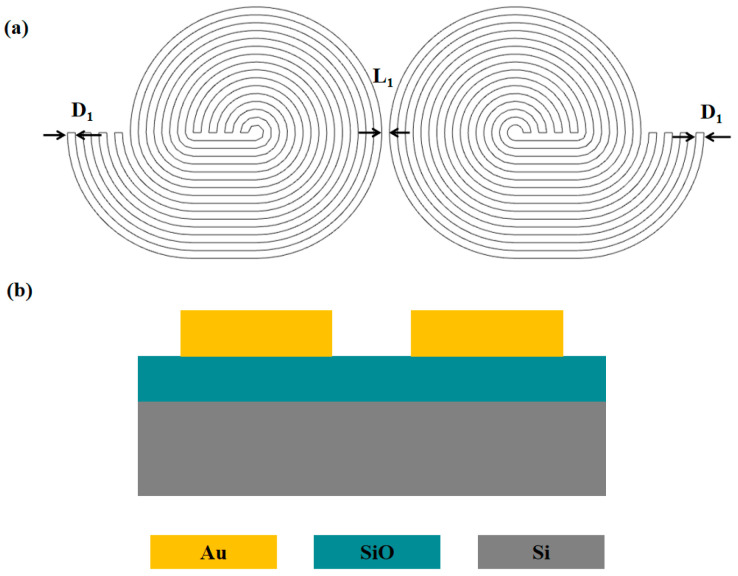
Schematic of the DEP device (**a**) helical forked electrode design. Four electrodes are arranged in an alternating circular pattern. All electrodes have a width of D1, and the distance between the two sets of electrodes is L1. When AC voltage is applied to the electrodes, the oligos in the aqueous solution on the electrodes experience sufficient DEP force to induce movement. (**b**) Side view of the single-plane DEP structure.

**Figure 7:**
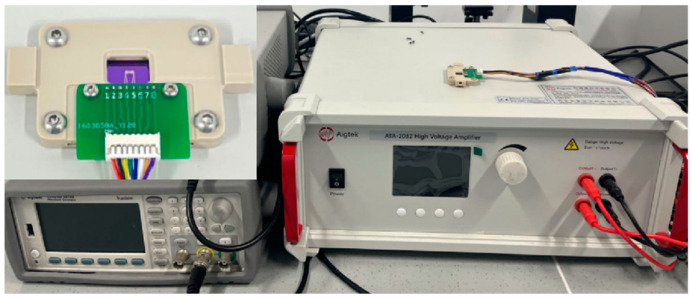
Microfluidic ink cartridge containing the DEP chip, along with the signal generator and high-voltage amplifier equipment for applying DEP forces.

We fabricated a chip, as shown in Figure 6, with integrated chemical modifications. This proof-of-concept design, outlined in Figure 6a, specifies the electrode width w, the distance between electrodes d, and the distance between electrode sets D. The substrate electrodes were patterned on a silicon wafer using plasma-enhanced chemical vapour deposition and magnetron sputtering etching, with an oxide layer and a gold layer both measuring 200 nm in thickness. A customized fixture, depicted in Figure 7, was used to assemble the chips for DEP functionality. AC power application was facilitated through the use of signal generators and high-voltage amplifiers.

### 4.4. Timing

The timing was established by conducting preliminary experiments to identify the duration required for optimal particle movement and assembly. We focused on ensuring that the DNA or particles were exposed to the DEP field long enough to achieve controlled manipulation but not so long that they would experience excessive heating or other side effects. The timings used in our study were based on these preliminary observations and were selected to allow for effective positioning and assembly of DNA.

### 4.5. Voltage/Frequency Pairing

The voltage and frequency pairing was selected to maximize DEP force while minimizing the risk of thermal damage to the samples. We performed a series of tests to evaluate how different voltage and frequency combinations affected the particle movement and assembly efficiency. The selected pairing represents a balance between strong DEP forces, which are essential for controlling the DNA positioning, and a frequency that ensures the DEP force is both effective and stable.

These parameters were optimized through trial and error in our experimental setup, with the aim of achieving efficient, controlled DNA manipulation and assembly under the specific conditions of our system.

### 4.6. Transporting Oligos

In the design of DEP electrodes, the spacing between electrodes is pivotal for oligos manipulation. For nano-sized oligo, reliable movement requires adjacent electrodes to be sufficiently close. On our platform, designed to assist trans-electrode oligos movement in a microfluidic system, the spacing between electrodes—ranging from 0.5 to 2 times the width of the electrode—ensures directional movement of oligos by DEP.

## Data Availability

The original contributions presented in the study are included in the article/Supplementary Material, further inquiries can be directed to the corresponding author.

## Supplementary Materials

The following supporting information can be downloaded at: https://www.mdpi.com/article/10.3390/mi16010076/s1, Table S1. Oligonucleotide sequences (5′→3′)and Primer for gene synthesis. Movie S1. Manipulate oligos by DEP.
