# Supplementary material for: On-Chip DNA Assembly via Dielectrophoresis
Source: Micromachines (Basel). 2025 Jan 11;16(1):76. doi: 10.3390/mi16010076 (PMC11767341; doi:10.3390/mi16010076)
Supplement: Supplementary file 1 [file micromachines-16-00076-s001.zip › Supplementary Information.pdf]

Supplementary Information for

"On-chip DNA Assembly via Dielectrophoresis"

Xichuan Rui, Lin-Sheng Wu, Xin Zhao*

State Key Laboratory of Radio Frequency Heterogeneous Integration (Shanghai Jiao Tong University), Shanghai, China

* To whom correspondence should be addressed:
* Xin Zhao: E-mail: xinzhao@sjtu.edu.cn

Table S1. Oligonucleotide sequences (5'→3')and Primer for gene synthesis.

Our oligonucleotide sequences (5'→3') and primer were purchased from Guiji Life Sciences, China. For analysis of the reserved complementary sequences, the sequences were matched using the primer information. The sequencing results were aligned and statistically analyzed using SnapGene.

Sequences name	Oligonucleotide sequences (5'→3')
1-F	GTGCATCACGTTGCGTGGAATGCAGCCACTGAATACTTTACG TCCATTTCGTCCAAGTTGGAT
1-R	CCTCATCCAACTTGGACGAAATGGACGTAAAGTATTCAGTG GCTGCATTCCACGCAACGTGATGCAC
2-F	GAGGCACCGTTGCCTCCGTCCAAATCATCAAACAGCTACAAT TACAAGGTGAAACGACCACTG
2-R	ATCGCAGTGGTCGTTTCACCTTGTAATTGTAGCTGTTTGATG ATTTGGACGGAGGCAACGGTG
3-F	CGATCCAAGTCGACAGGGAACATTGCACAGCTATCCACTGA ACAATATAACGAAATAATGGCA
3-R	CCAGTGCCATTATTTCGTTATATTGTTCAGTGGATAGCTGTG CAATGTTCCCTGTCGACTTGG
4-F	CTGGCAAGGGGGAACTTTGACATCTCGCTACTACCGAGTGAT GCAGAGATTTTGATATTCTCG
4-R	AGAACGAGAATATCAAAATCTCTGCATCACTCGGTAGTAGC GAGATGTCAAAGTTCCCCCTTG
5-F	TTCTTGCACCCCAAAGACGTGTTGAACTTCACTTGTACCAAT CGAGCAGGGAGGAGAATGCTG

5-R	CATCCAGCATTCTCCTCCCTGCTCGATTGGTACAAGTGAAGTTCAACACGTCTTTGGGGTGCA
6-F	GATGATGGCTTAGCTATTGCGAATGAAAAGACTGAAAGTCAACATGATAAAGCGGGGGATTCG
6-R	ACGCCGAATCCCCCGCTTTATCATGTTGACTTTCAGTCTTTTCATTCGCAATAGCTAAGCCAT
7-F	GCGTTGTTGATTTGGAAAGCACTTTTTCAACGAGATTTTGCGTGGGTGCTGTCTGATTGGAAG
7-R	CCATCTTCCAATCAGACAGCACCCACGCAAAATCTCGTTGAAAAAGTGCTTTCCAAATCAACA
8-F	ATGGGGAAGGAGGCGTTTGCGAGATCGATGATAAAATATAAACTGCGAGGGTGGCAGGAAGAA
8-R	TTCTTCCTGCCACCCTCGCAGTTTATATTTTATCATCGATCTCGCAAACGCCTCCTTCC
Forward primer	ATC ACG TTG CGT GGA ATG CA
Reverse primer	TTC TTC CTG CCA CCC TCG

Supplementary Movies

Movie S1. Manipulate oligos by DEP.
